# Hematopoietic Stem Cell Transplantation for C1q Deficiency: A Study on Behalf of the EBMT Inborn Errors Working Party

**DOI:** 10.1007/s10875-024-01819-1

**Published:** 2024-10-29

**Authors:** Helena Buso, Etai Adam, Peter D. Arkwright, Sagar Bhattad, Amir Ali Hamidieh, Maryam Behfar, Alexandre Belot, Sarah Benezech, Alice Y. Chan, Yanick J. Crow, Christopher C. Dvorak, Aisling M. Flinn, Urvi Kapoor, Arjan Lankester, Masao Kobayashi, Risa Matsumura, Hadi Mottaghipisheh, Satoshi Okada, Marie Ouachee, Nima Parvaneh, Stalin Ramprakash, Prakash Satwani, Samin Sharafian, Clément Triaille, Robert F. Wynn, Nasim Movahedi, Vahid Ziaee, Eleri Williams, Mary Slatter, Andrew R. Gennery

**Affiliations:** 1 Department of Medicine (DIMED), University of Padova, Padua, Italy; 2 Paediatric Haematopoietic Stem Cell Transplant Unit, Great North Children’s Hospital, Newcastle Upon Tyne, NE1 4LP UK; 3 Sheba Medical Center, The Edmond and Lily Safra Children’s Hospital, Ramat Gan, Israel; 4 Lydia Becker Institute of Immunology and Inflammation, University of Manchester, Manchester, UK; 5 Division of Paediatric Immunology and Rheumatology, Department of Paediatrics, Aster CMI Hospital, Bengaluru, India; 6 Pediatric Cell and Gene Therapy Research Center, Gene, Cell & Tissue Research Institute, Tehran University of Medical Sciences, Tehran, Iran; 7 Department of Paediatric Rheumatology, Femme-Mère-Enfant Hospital, HCL, Lyon, France; 8 Institute of Hematology and Pediatric Oncology, 69008 Lyon, France; 9 Division of Pediatric Allergy, Immunology, and Blood and Marrow Transplant, UCSF Benioff Children’s Hospital, University of California San Francisco, San Francisco, CA USA; 10 Laboratory of Neurogenetics and Neuroinflammation, Imagine Institute, INSERM UMR1163, Paris, France; 11 MRC Human Genetics Unit, Institute of Genetics and Cancer, University of Edinburgh, Edinburgh, UK; 12 Department of Pediatric Immunology, Children’s Health Ireland at Crumlin, Dublin, Ireland; 13 Division of of Pediatrics Haematology, Oncology and Stem Cell Transplant, Children’s Hospital New York-Presbyterian, Columbia University, 161 Fort Washington, Irving 7, New York, NY 10032 USA; 14 Department of Pediatrics, Willem-Alexander Children’s Hospital, Leiden University Medical Center, Leiden, The Netherlands; 15 Department of Pediatrics, Hiroshima University Hospital, 1‑2‑3 Kasumi, Minami‑ku, Hiroshima, 734‑8551 Japan; 16 Hematology Research Center, Shiraz University of Medical Sciences, Shiraz, Iran; 17 Division of Allergy and Clinical Immunology, Department of Pediatrics, Tehran University of Medical Sciences, Tehran, Iran; 18 Aster International Institute of Oncology, Aster CMI Hospital, Bangalore, India; 19 Department of Allergy and Clinical Immunology, Mofid Children’s Hospital, Shahid Beheshti University of Medical Sciences, Tehran, Iran; 20 Pôle de Pathologies Rhumatismales Systémiques Et Inflammatoires, Institut de Recherche Expérimentale Et Clinique, Université Catholique de Louvain, Brussels, Belgium; 21 Pediatric Immunology and Rheumatology Division, Department of Pediatrics, CHU Sainte-Justine, University of Montreal, Montreal, QC Canada; 22 Department of Paediatric Haematology & Oncology, Royal Manchester Children’s Hospital, Manchester, UK; 23 Golestan Rheumatology Research Center (GRRC), Golestan University of Medical Sciences, Gorgan, Iran; 24 Pediatric Rheumatology Research Group, Rheumatology Research Center, Tehran University of Medical Sciences, Tehran, Iran; 25 Department of Pediatrics, Tehran University of Medical Sciences, Tehran, Iran; 26 Translational and Clinical Research Institute, Newcastle University, Newcastle Upon Tyne, UK

**Keywords:** Allogeneic HSCT, C1q deficiency, SLE

## Abstract

C1q deficiency is a rare inborn error of immunity characterized by increased susceptibility to infections and autoimmune manifestations mimicking SLE, with an associated morbidity and mortality. Because C1q is synthesized by monocytes, to date, four patients treated with allogeneic HSCT have been reported, with a positive outcome in three. We conducted an international retrospective study to assess the outcome of HSCT in C1q deficiency. Eighteen patients, fourteen previously unreported, from eleven referral centres, were included. Two patients had two HSCTs, thus 20 HSCTs were performed in total, at a median age of 10 years (range 0.9—19). Indications for HSCT were autoimmune manifestations not controlled by ongoing treatment in seventeen, and early development of MALT lymphoma in one patient. Overall survival (OS) was 71% and event-free survival was 59% at two years (considering an event as acute GvHD ≥ grade III, disease recurrence and death). In eleven patients HSCT led to resolution of autoimmune features and discontinuation of immunosuppressive treatments (follow-up time range 3–84 months). Five patients died due to transplant-related complications. Patients with a severe autoimmune phenotype, defined as neurological and/or renal involvement, had the worst OS (40% vs 84%; *p* = 0.034). Reviewing data of 69 genetically confirmed C1q deficient patients, we found that anti-Ro antibodies are associated with neurologic involvement, and anti-RNP and anti-DNA antibodies with renal involvement. In conclusion, HSCT may be a valid curative option for C1q deficiency, but careful selection of patients, with an accurate assessment of risk and benefit, is mandatory.

**Supplementary Information:**

The online version contains supplementary material available at 10.1007/s10875-024-01819-1.

## Introduction

C1q deficiency is a rare autosomal recessive inborn error of immunity (IEI) caused by biallelic mutations in one of the three C1q genes (*C1QA*, *C1QB*, and *C1QC*) [1]. C1q is the first molecule of the classical complement pathway and plays a major role in the innate immune response, and clearance of immune complexes and apoptotic cells [2–4]. The first case of C1q deficiency was reported in 1978, describing a 10-year-old boy with recurrent skin lesions and chronic infections [5].

Since then, more cases have been described with a variable clinical phenotype that ranges from severe infections (e.g. meningitis) to autoimmune manifestations, mirroring the complex physiological role of C1q [6, 7]. Autoimmunity was the most prominent finding in a description of the clinical manifestations of 71 C1q deficient patients, where more than 75% of cases fulfilled the classification criteria for systemic lupus erythematous (SLE) or a lupus-like syndrome (according to the 1997 American College of Rheumatology criteria [8]) with a great number of severe cases with renal (31%) and central nervous system (CNS) involvement (20%) [9]. Of note, in comparison with sporadic SLE, C1q deficiency is characterized by an earlier disease onset, more extensive cutaneous involvement and a different autoantibody profile with a lower frequency of anti-dsDNA antibodies [9].

As described in sporadic SLE, hyperactivation of interferon-alpha (IFN-α) signalling sustains the autoimmune response [10, 11]. Indeed, C1q is required to inhibit IFN-α production by plasmacytoid dendritic cells [12], and thus the absence of C1q leads to IFN-α dysregulation. For that reason, C1q deficiency has been suggested to be a Mendelian type I interferonopathy [13]. Management includes corticosteroids and immunosuppressive drugs to control the immune dysregulation, combined with antibiotic prophylaxis when needed. Administration of C1q through fresh frozen plasma (FFP) has shown some effectiveness in attenuating disease features but does not provide a definitive and permanent treatment [14–16]. Unfortunately, in some patients, despite the use of multiple therapies, the disease remains uncontrolled with consequent high disease burden, organ damage and mortality at a young age [6]. As C1q is mainly produced by monocytes (in contrast to other complement proteins that are mainly produced by hepatocytes), it was hypothesised that allogeneic hematopoietic stem cell transplantation (HSCT) could be a definitive treatment for this disorder [17]. In C1q-knockout mice, the transplantation of stem cells from wild-type animals restored C1q levels with consequent resolution of autoimmunity [18, 19].

To date, four patients with C1q-deficiency treated by HSCT have been reported. In three, HSCT led to normalization of complement activity and consequent disease resolution. Unfortunately, one patient died from HSCT-related complications [20–22]. Considering the variable clinical presentation with different patterns of disease severity, more information about HSCT indications and efficacy for C1q deficiency is needed.

Here, we describe fourteen previously unreported patients with C1q deficiency who were treated with HSCT, and we provide an update on two previously published cases. Finally, we review the main clinical features, genetic mutations, and anti-nuclear antibody (ANA)-specificity of our cohort and of previously described genetically confirmed C1q deficient patients, to identify possible markers of disease severity.

## Methods

### Data Collection of Transplant Patients

A retrospective data collection of clinical, laboratory and immunological features from written and electronic medical records of C1q deficient patients treated with HSCT across eleven different referral centres in the world was performed. Patients were identified through the Center for International Blood and Marrow Transplant Research (CIBMTR), Primary Immune Deficiency Treatment Consortium (PIDTC), European Bone Marrow Transplant (EBMT) and Stem Cell Transplantation for Immunodeficiencies in Europe (SCETIDE) registries and personal contact with physicians who had transplanted patients. A review, and when possible, an update, of already reported cases was performed. For all patients, families had given prior written consent.

Patients were classified as 'severe autoimmune phenotype' based on the presence of significant extracutaneous involvement (neurological and/or renal disease).

### Literature Review

We retrieved data on 77 genetically confirmed C1Q deficient patients from the recent article by Triaille and colleagues [23] identified by a Pubmed search with the term “C1Q deficiency” for the period from December 2011 to January 2024, and retrieving cases described before January 2011 from systematic reviews conducted by Schejbel and colleagues [1], and Jlajla and colleagues [24].

For each patient data on gene mutations, anti-nuclear antibody specificity and main clinical manifestations categorised as major infections, mucocutaneous, CNS, and renal involvement were collected. We defined CNS involvement as a non-infectious inflammatory/degenerative process, excluding meningitis and other infectious events, and including CNS vasculitis, myelopathy, cerebral atrophy and basal ganglia calcification. We defined renal involvement as lupus-like glomerulonephritis. Data on more rare clinical features were not collected. The CNS and/or renal involvement was considered as a marker of severe disease phenotype.

In the subgroup of patients with available details on molecular lesion and specific autoantibody profile we investigated whether specific gene mutations and/or autoantibody subsets were associated with severe disease phenotype.

### Statistical Tests

Quantitative variables were summarised as medians with ranges, and categorical variables as numbers and percentages of the group. The overall survival (OS) and the event free survival (EFS) were captured using the Kaplan–Meier method. We considered as an event: acute (a)GvHD ≥ grade III, disease recurrence due to loss of chimerism, and death. The Log-rank test was used to compare OS and EFS between patients with mild and severe disease phenotype. Chi-squared testing was used to assess possible association between defined gene mutations and presence of specific autoantibody with different clinical manifestations.

## Results

### Features of HSCT Population

The study included 18 C1q deficient patients from 11 referral centres, of whom 14 were previously unreported. In addition, two of four previously reported cases (P15, P16, P17, P18) were updated (*Data summarized in* Table 1).

**Table 1:** Patient characteristics and disease course before HSCT

Pt Sex	Age at disease onset	C1q gene mutation	Infections	Mucocutaneous involvement	Autoimmune cytopenias	Neurologic Involvement	Other	Auto-antibodies	Therapy
P1 F	3 y	C1QA c.622C > T p.Gln208X	Respiratory infections	Malar rash	No	No	Hypothyroidism	ANA, anti-Ro, anti-La, anti-Sm, anti-TPO	HCQ, topical TACRO, Azithromycin
P2 F	Early Infancy	C1QA c.127G > A p.Gly43Arg	Respiratory and mucocutaneus infections	Malar rash, oral ulcers, cutaneous vasculitis, urticarial rash	Neutropenia	No	Recurrent fever, splenomegaly, lymphadenitis, arthralgia	ANA, anti-Ro, anti-neutrophil, RF	Steroids, Sirolimus, RTX, MMF, G-CSF, FFP, Azithromycin, Penicillin
P3 F	Infancy	C1QA p.Gln208X	S. pneumoniae sepsis. Facial HSV	Malar rash, oral ulcers, alopecia, cutaneous vasculitis	No	CNS vasculitis	Recurrent fever, weight lost, Kikuchi lymphadenitis, angiitis of splanchnic -hepatic vasculature	Negative	Steroids, CYC, RTX, IFX, AZA, HCQ, IVIG monthly, Valaciclovir and Co-trimoxazole
P4 M	18 m	C1QC c.271G > T p.Gly91X	No	Malar rash, palmar/plantar erythema, oral ulcers	No	No	Arthralgia	ANA, anti-Ro, anti-Sm	Steroids, HCQ, FFP, amoxicillin
P5 F	4 y	C1QB p.Gly244Arg	No	Malar rash, discoid lupus, oral ulcers, alopecia, panniculitis, cutaneous vasculitis	Leukopenia	No	Constitutional symptoms	ANA, anti-Ro, anti-Sm, anti-RNP, anti-β2 GP	Steroids,high dose IVIG, MMF, AZA, HCQ, RTX, topical steroids and topical TACRO
P6 M	9 y	C1QB c.268G > A; p.Gly90Ser	No	Parotitis, Sjogren	Leukopenia	No	MALT lymphoma	ANA, anti-Ro, anti-La	Steroids, FFP
P7 F	2 y	C1QA c.44delT	Otitis media	Oral ulcers, alopecia, hyperpigmented lichenificated skin	Thrombocytopenia	Myelopathy, spasticity of lower limbs	Pulmonary hypertension, glomerulonephritis (class IV LN)	ANA	CYC, Rituximab, MMF, TACRO
P8 F	18 m	C1QA c.622C˃T p.Gln208X	No	Pustular facial skin lesions	No	Diffuse cerebral atrophy, development delay	No	ANA, anti-RNP, anti-Sm, anti-Ro	Steroids, HCQ, FFP, clindamycin, fluconazole, acyclovir
P9 F	4 y	C1QC c.611C > T p.Ser204Leu	Chicken pox	Malar rash, oral ulcers, alopecia	Leukopenia	No	Musculoskeletal involvement	ANA, RF, anti-β2 GP	Steroids, HCQ
P10 M	5 y	C1QA c.622C˃T p.Gln208X	No	Urticarial rash	No	No	Musculoskeletal involvement, constitutional symptoms	ANA, anti-dsDNA	Steroids, HCQ, Colchicine
P11 F	9 m	C1QA c.622C˃T p.Gln208X	No	Malar rash, discoid lupus, oral ulcers, alopecia	No	No	Musculoskeletal involvement constitutional symptoms	ANA, anti-Ro anti-Sm, RF	Steroids,, HCQ, AZA
P12 M	1 m	C1QA (precise mutation not known)	Oral thrush	Malar rash, urticarial vasculitis.	No	No	No	ANA, anti-Ro, anti-La, RF	Steroids
P13 F	9 m	C1QA c.101G > A p.Gly34Glu	No	Malar rash, oral ulcers	No	No	No	ANA, anti-Sm anti-DNA, anti-RNP anti-cardiolipin, anti-β2 GP	Steroids, HCQ, MMF, Baracitinib
P14 M	1 y	C1QB c.187 + 1G > T	No	Malar rash	Thrombocytopenia	No	No	RF	Steroids, FFP
P15 F [22]	4 y	C1QB c.187 + 1G > T	No	Malar rah, oral ulcers	No	No	Fever	ANA, anti-RNP, anti-Sm	Steroids, MMF, FFP
P16 M [21]	3 y	Not tested	Bacterial meningitis	Malar rash	No	Cerebral vasculitis	No	Anti-Ro, anti-cardiolipin	Steroids, CYC, RTX, ATB prophylaxis
P17 M [20]	15 m	C1QA Gln208X	No	Skin lesions	No	Cerebral vasculitis	Glomerulonephritis, fever	ANA, anti-Rnp, anti-Ro	Steroids, RTX, FFP
P18 F [20]	8 m	C1QA Trp216X	Bacterialmeningitis with septicaemia, respiratory infections	Alopecia, discoid lupus, oral ulcers	Leukopenia	No	Uveitis	ANA, anti-Ro	Steroids, HCQ, FFP, penicillin

*ANA* anti-nuclear antibodies, *ATB* antibiotic, *AZA* Azathioprine, *CNS* central nervous system, *CYC* cyclophosphamide, *F* female, *FFP* Fresh Frozen Plasma, *G-CSF* granulocyte colony-stimulating factor, *HCQ* Hydroxychloroquine, *HSV* herpes simplex virus, *IFX* infliximab, *IVIG* intravenous immunoglobulins, *y* years, *M* male, *m* months, *MMF* mycophenolate mofetil, *RF* rheumatoid factor, *RTX* rituximab, *TACRO* tacrolimus

Eleven (61%) patients were female. The median age at disease onset was 2.5 years (range, 0.5 months – 9 years). The C1q genetic defect was determined in 17 patients, with mutations in *C1QA* in 11, *C1QB* in 4 and *C1QC* in 2 patients. The most frequent variant was Gln208X in the *C1QA* gene, present in 6 patients. P14 and P15 were siblings with the same homozygous mutation (c.187 + 1G > T).

All patients demonstrated an autoimmune/autoinflammatory phenotype with a broad spectrum of clinical manifestations: mucocutaneous involvement was reported in all 18 patients in combination with cytopenia in 7 cases (39%), neurologic involvement in 5 cases (28%) and glomerulonephritis in 2 cases (11%). Three patients (17%) had lymphoproliferation-associated disorders, such as lymphadenitis and splenomegaly, and one patient (P6) developed mucosa-associated lymphoid tissue (MALT) lymphoma in the context of Sjogren syndrome. Eight (44%) patients had exhibited symptoms including recurrent fever, arthralgia, and weight loss.

All these disease manifestations resulted in a significant disease burden, that required use of steroids and/or various immunosuppressive treatments leading to important side effects such as osteonecrosis, hypertension, and growth retardation. In 8 patients (44%) FFP infusions were given in conjunction with immunosuppressive drugs. In 6 cases (33%) severe and/or recurrent infections were reported and 7 patients (39%) were receiving antibiotic prophylaxis. Of note, only three patients had history of severe infections with one case of S. pneumoniae sepsis (P3) and two cases of meningitis (P16, P18).

### Markers of Disease Severity

We reviewed 89 patients with genetically confirmed C1Q deficiency (including 14 previously unreported cases from our cohort). To the cohort of 77 patients C1Q analysed by Triaille and colleagues [23], (which already included P2, P3, P15, P17, P18), we have added 12 genetically confirmed C1q deficient patients from our cohort (P1, P4, P5, P6, P7, P8, P9, P10, P11, P12, P13, P14).

Variants were seen in *C1QA*, *CQ1B*, and *CQ1C* in 56%, 12%, and 32% of the 89 patients, respectively.

The most frequent mutations were Gln208X in *C1QA* reported in 31 cases (35%), Arg69X in *C1QC* reported in 8 cases (9%) and Gly34Arg in *C1QC* in 8 cases (9%).

In 69 patients, mutation data were available, specific autoantibodies were tested and main clinical manifestations were recorded. In this subgroup of patients, we investigated if specific genotypes and/or autoantibody subset were associated with an autoimmune-driven CNS or renal involvement.

As in our 18 transplant patients, in this larger cohort the mucocutaneous involvement was the most common manifestation, reported in 62 (90%), and a significant percentage of patients 21 (30%) had neurologic involvement. On the other hand, patients with renal involvement and severe infections were more frequent in this cohort, respectively 16 (23%) and 21 (30%).

We found no association between the three most frequent gene variants (Gln208X, Arg69X, Gly34Arg) and different clinical manifestations*.* Anti-nuclear antibody (ANA) titres were positive in 65 (94%) of patients, with anti-Ro specificity in 37/69 (54%), anti-Sm in 32/65 (49%), anti-RNP in 22/65 (34%) and anti-DNA in 13/65 (20%).

**Fig. 1:**
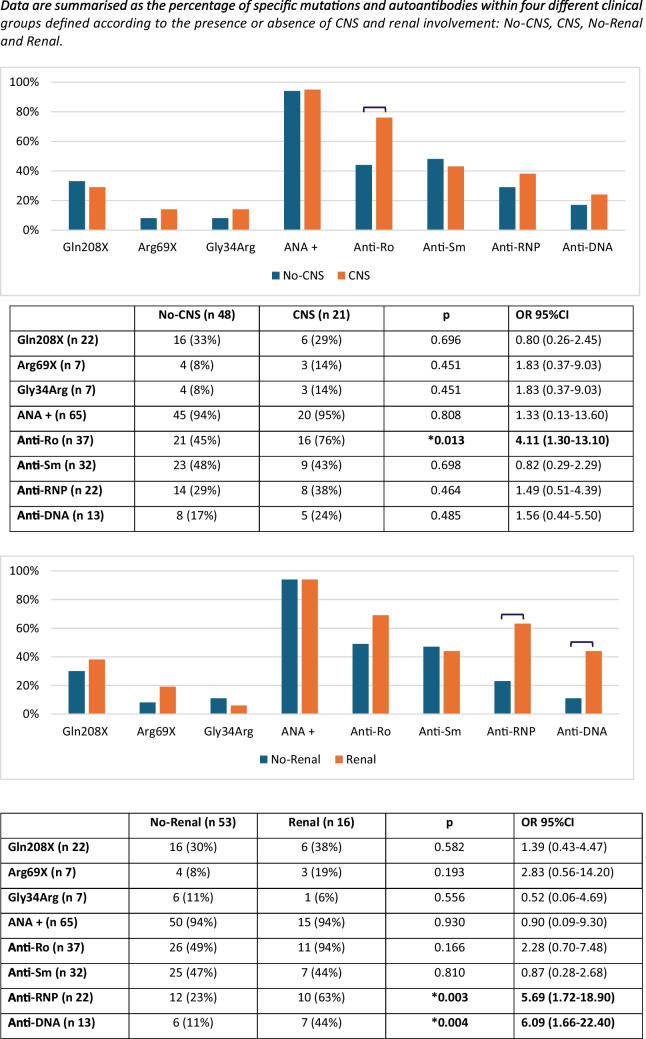
Possible markers of disease severity. Data are summarised as the percentage of specific mutations and autoantibodies within four different clinical groups defined according to the presence or absence of CNS and renal involvement: No-CNS, CNS, No-Renal and Renal

Analysing different autoantibody specificities, we found that anti-Ro associated with CNS involvement (OR 4.11; IC95% 1.30–13.10) and anti-RNP and anti-DNA with renal involvement (respectively OR 5.69; IC95% 1.72–18.9 and OR 6.09; IC95% 1.66–22.40) (Fig. 1).

### HSCT Details and Outcome

**Fig. 2:**
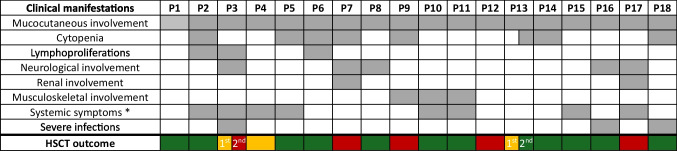
Clinical features and HSCT outcome. Gray squares represent the presence of a clinical feature/phenotype. Green squares indicate that patients survive after HSCT. Yellow squares indicate that patients had a graft failure. Red squares indicate that patients died after HSCT. For patients P3 and P13 that had two HSCT the outcome of both transplants is indicated. * Recurrent fever, arthralgia and weight loss

Two patients (P3, P13) had two HSCTs, thus 20 HSCTs were performed in total (Table 2). In 17/18 patients the indication for HSCT was the persistence of symptoms despite ongoing treatments. P6 underwent HSCT because of high-risk disease with early development of MALT lymphoma on a background of Sjogren syndrome, quiescent at the time of HSCT (Fig. 2*summarises the main baseline features and the overall HSCT outcome*).

**Table 2:** HSCT details

Pt n-HSCT	Age at HSCT	Lansky Score at HSCT	Donor Stem Source	NC (10^8^ /Kg)	CD34 + (10^6^ /Kg)	CD3 + (10^6^ /Kg)	Conditioning and Serotherapy	GVHD drugs	Main HSCT complications	Neu	Pla	Last Chimerism	Outcome and FU-time
P1 n-1	9.7 y	90	MMUD (9/10) MMA PBSC TCR α-β depleted	7.2	7.0	22	RTC: Treosulfan Fludarabine Thiotepa Rituximab ATG	CSA MMF	Acute GVHD skin (grade II), HHV6 viraemia	+ 12	+ 14	CD3 100% CD15 100% CD19 100%	Alive (36 m) Resolution of malar rash, persistence of infection and hypothyroidism Ongoing azithromycin
P2 n-1	15.8 y	90	MUD (10/10) PBSC	9.6	4.8	340	RTC: Treosulfan Fludarabine Thiotepa Alemtuzumab	CSA MMF	No	+ 17	+ 17	CD3 100% CD15 100% CD19 100%	Alive (3 m) Resolution of symptoms No treatments
P3 n-1	15.2 y	80	MMRD MMA (9/10) BM	1.9	0.7	29	RIC: Treosulfan Fludarabine Alemtuzumab	CSA MMF	Acute GVHD skin (grade I), possible VOD, CMV and HHV6 viraemia, oral candida, haemorrhagic cystitis (BK), E. Coli UTI	+ 26	+ 32	CD3 8% CD15 3% CD19 0%	Slipping Chimerism since day + 41 After 27 months CNS vasculitis treated with steroids, RTX and MMF
P3 n-2	19.1 y	70	MUD (10/10) PBSC	12.5	7.5	490	RTC: Treosulfan Fludarabine Thiotepa Alemtuzumab	CSA MMF	CMV viraemia, engraftment syndrome, CSA-neurotoxicity, endothelial alveolar haemorrhage, PCJ and Aspergillus pneumonia	+ 10	Not reach	Total 100%	Died (day + 32) Aspergillus Pneumonia
P4 n-1	2.5 y	90	MSD (12/12) BM	3.5	3.0	29	RIC: Treosulfan Fludarabine ATG	CSA MMF	CMV viraemia	+ 9	+ 14	/	Alive (21 m) Secondary graft failure (Chimerism < 20% after 14 months) Recurrence of symptoms (malar rash, oral ulcers), HCQ and FFP restarted
P5 n-1	7.5 y	100	MMUD (9/10) MMA BM RBC depletion	4.5	/	/	MAC: Melphalan Fludarabine Thiotepa Alemtuzumab	TACRO MTX Steroid	Adenovirus viraemia	+ 19	+ 18	CD3 100% CD15 100% CD19 100%	Alive (7 y) Resolution of symptoms No treatments
P6 n-1	13.5 y	90	MUD (10/10) BM	2.1	1.6	/	RTC: Treosulfan Fludarabine Thiotepa ATG	CSA MTX	Acute GVHD skin-eyes (grade II), bronchiolitis obliterans,hypergonadotropic hypogonadism	+ 28	+ 19	CD3 100% CD15 100% CD19 100%	Alive (4 y) Resolution of symptoms No treatments
P7 n-1	13.6 y	40	MRD (10/10) BM	21	3.46	/	MAC: Busulfan Fludarabine Thiotepa ATG	TACRO MMF	Acute GVHD gut (grade IV), CMV viraemia, MRSA pneumonia, ARDS, TMA	+ 15	+ 25	/	Died (day + 87) MOF in TMA, acute GVHD of gut and ARDS
P8 n-1	3.3 y	40	MRD (10/10) PBSC	8.5	5	360	RIC: Melphalan Fludarabine ATG	CSA MPS	Acute GVHD skin (grade I), CMV viraemia	+ 11	+ 10	Total 99%	Alive (13 m) Resolution of symptoms No treatments
P9 n-1	16 y	30	MSD (10/10) PBSC	8.2	9	356	RIC: Melphalan Fludarabine ATG	CSA MPS	Acute GVHD gut (grade III), CMV viraemia, haemorrhagic cystitis (BK), renal failure, Aspergillus pneumoniae	+ 10	+ 10	Total 95%	Died (5 m) Encephalopathy in idiopathic hyperammonaemia
P10 n-1	5.8 y	50	MSD (10/10) PBSC	10.3	7.3	229	RIC: Melphalan Fludarabine ATG	CSA MTX	Acute GVHD skin (stage III), gut (stage IV), CMV viraemia, haemorrhagic cystitis (BK), Aspergillus pneumonia, Staphylococcus bacteriemia	+ 14	+ 14	Total 99%	Alive (21 m) Resolution of symptoms No treatments
P11 n-1	4.7 y	40	MRD (10/10) PBSC	6.7	10.4	252	RIC: Melphalan Fludarabine ATG	CSA	PRESS	+ 11	+ 9	CD3 100% CD15 100% CD19 100%	Alive (6 m) Resolution of symptoms No treatments
P12 n-1	0.9 y	100	MMUD (9/10) MMA PBSC CD34 selection	0.11	10.6	/	RTC: Busulfan, Fludarabine, Alemtuzumab	TACRO	Adenovirus viremia, aerococcus bacteremia, VOD, IPS, CMV pneumonia	+ 10	Not reach	Total 100%	Death (3 m) Respiratory failure in CMV pneumonia
P13 n-1	3.6 y	/	MUD (10/10) BM RBC depletion	4.6	2.5	33	RTC: Busulfan Fludarabine ATG	CSA, MMF	Acute GVHD of skin (stage III) and gut (stage II), autoimmune pancytopenia, BK viremia, E. Coli septic shock	+ 23	+ 26	/	Alive (21 m) Secondary graft failure Day + 146 Development of severe autoimmune anemia requiring immunosuppressors
P13 n-2	4.3	/	MUD (10/10) PBSC RBC depletion	13.9	5.7	457	RTC: Treosulfan Fludarabine Thiotepa Alemtuzumab	CSA, MMF	Acute GVHD skin (grade II), AIHA	+ 15	+ 10	Total 99.7%	Alive (8 m) Development of post-HSCT haemolytic autoimmune anaemia, requiring CSA, steroids and RTX
P14 n-1	10.3 y	80	MUD (8/8) BM	3.5	5.0	/	RIC: Melphalan Fludarabine TBI ATG	TACRO MTX	Acute GVHD skin (grade I), engraftment syndrome	+ 12	+ 16	Total 100%	Alive (24 m) Resolution of symptoms No treatments
P15 n-1 [22] Update	12 y	80	MMUD (7/8) BM	3.1	1.8	/	RIC: Melphalan Fludarabine TBI ATG	TACRO MTX	Acute GVHD skin (grade I), engraftment syndrome	+ 16	+ 22	Total 100%	Alive (5 y) Resolution of symptoms No treatments
P16 n-1 [21] Update	16 y	80	MSD (10/10) PBSC	/	10	/	RTC: Treosulfan Fludarabine Thiotepa Alemtuzumab	CSA MMF Steroid	EBV viremia	+ 14	+ 14	CD3 100% CD15 100% CD19 100%	Alive (5.5 y) Resolution of symptoms No treatments
P17 n-1 [20]	9 y	70	MUD (10/10) BM	4.5	2.5	/	MAC: Treosulfan Fludarabine Etoposide ATG	CSA MTX	Acute GVHD gut (grade II), EBV-PTLD	+ 36	/	CD3 99% CD14 99%	Died (4 months) MOF
P18 n-1 [20]	12 y	90	MSD (10/10) BM	9.8	10.1	/	RIC: Treosulfan Fludarabine ATG	CSA MTX	EBV-PTLD, VZV disease	+ 18	/	CD3 43% CD14 45% CD19 62%	Alive (33 m) Resolution of symptoms No treatments

*AIHA* autoimmune haemolytic anaemia, *ARDS* acute respiratory distress syndrome, *ATG* anti-thymocyte globulin, *BM* bome marrow, *CMV* cytomegalovirus, *CSA* Cyclosporine, *EBV* epstein-barr virus, *HHV6* herpes virus 6, *GVHD* graft versus host disease, *HCQ* hydroxychloroquine, *HSCT* hematopoietic stem cell transplantation, *IPS* idiopathic pneumonia syndrome, *MAC* myeloablative conditioning, *MMA* mismatch HLA-A, *MMDR* mismatch HLA-DR, *MSD* matched sibling donors, *MRD* matched related donors, *MUD* matched unrelated donors, *MMRD* mismatched related donor, *MMUD* mismatched unrelated donors, *MMF* mycophenolate mofetil, *MTX* methotrexate, *MOF* multiorgan failure, *MRSA* methicillin-resistant staphylococcus aureus, *MRI* magnetic resonance imaging, *PBSC* peripheral blood stem cells, *PJP* pneumocystis carinii pneumonia, *PTLD* post-transplant lymphoproliferative disorder, *RBC* red blood cells depletion, *RIC* reduced intensity conditioning, *RTC* reduced toxicity conditioning, TACRO tacrolimus, *TCR* T cell receptors, *TMA* transplant-associated thrombotic microangiopathy, *UTI* urinary tract infection, *VOD* veno-occlusive disease

The median age at HSCT was 10 years (range 0.9—19 years) with a median time between symptom onset and HSCT of 7.2 years (range 0.8 – 14 years). Different donors were used: 5 (25%) matched sibling donors (MSD), 3 (15%) matched related donors (MRD), 7 (35%) matched unrelated donors (MUD), 1 (5%) mismatched related donor (MMRD) and 4 (20%) mismatched unrelated donors (MMUD). The stem cell source was bone marrow in 10 cases (50%) and peripheral blood stem cells (PBSC) in the remaining cases. Different conditioning regimens were used, both myeloablative and reduced toxicity, based on Treosulfan in 10, Melphalan in 7 and Busulfan in 3 cases.

**Fig. 3:**
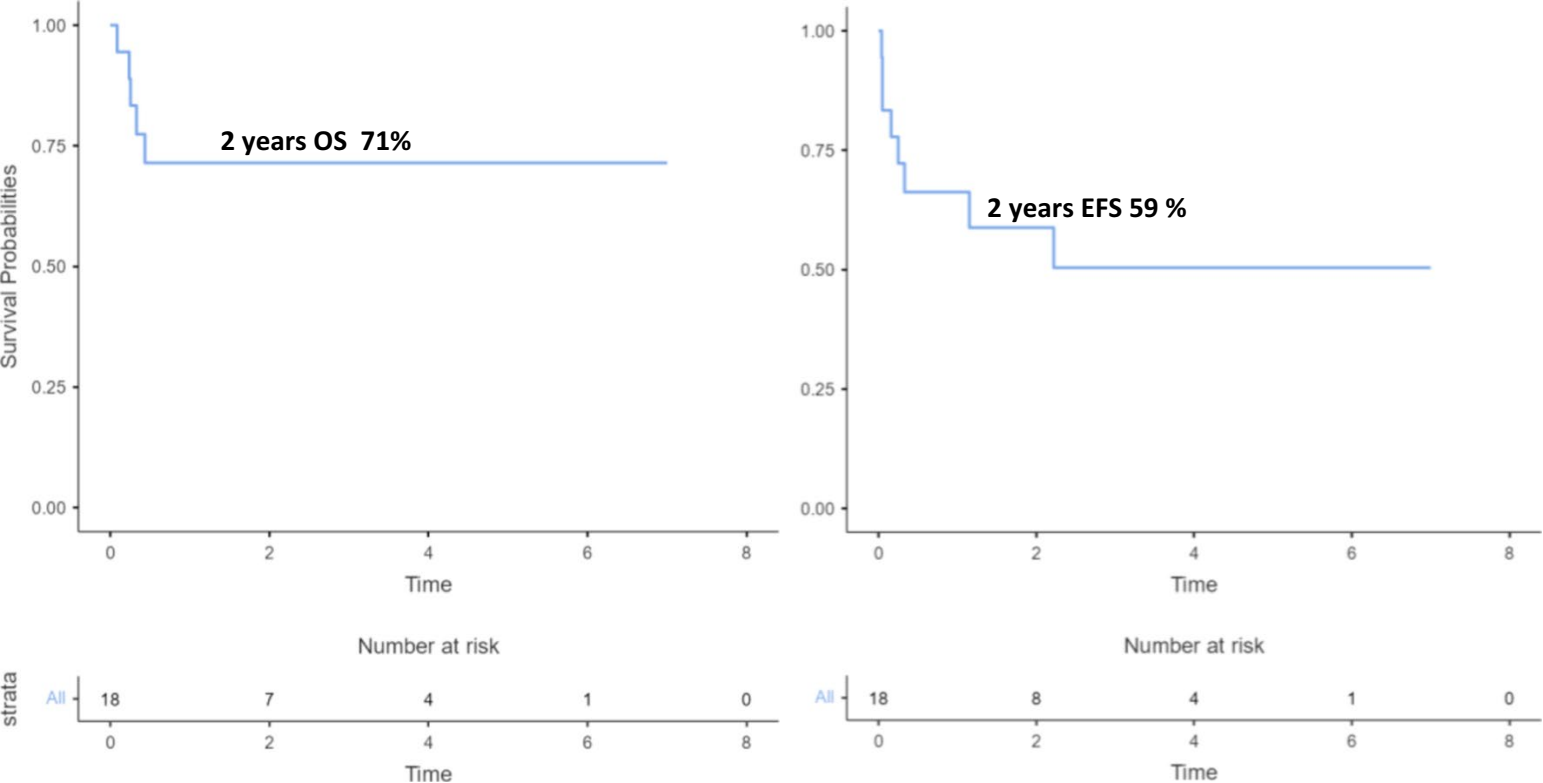
Overall survival (OS) and Event Free Survival (EFS) of the whole cohort. The overall survival at two years was 71% (95%CI 44–87%). For patients who had two HSCT, 2nd HSCT was considered as baseline. The event free survival at two years was 59% (95%CI 32–78%). Event: aGvHD ≥ grade III; disease recurrence due to loss of chimerism; death

All patients achieved neutrophil engraftment after a median of 15 days (range 9 – 36 days). The OS in the whole group was 71% (95%CI 44–87%) at 2 years and the EFS was 59% (95%CI 32–78%) at 2 years (Fig. 3 ).

Seven patients (39%) developed aGvHD of at least overall grade II, with involvement of skin in three (P1, P6, P13.2), gut in three (P7, P9 and P17) and both skin and gut in two patients (P10, P13.1). Of note, P13 developed aGvHD both after the first and the second HSCT. Only one patient (P6) reported chronic GvHD of the lung (bronchiolitis obliterans).

In 11 patients (61%), HSCT led to resolution of autoimmune features allowing for discontinuation of immunosuppressive treatment (median follow-up time since HSCT 33 months, range 3–84 months). In ten, there was a documented normalization of the function of the classical complement pathway (CH50) and/or of C1q level after HSCT (P10 had no available CH50 and C1q level post-HSCT) (Supplementary Table 1).

Ten patients demonstrated full donor chimerism at the time of last follow up and one (P18) had mixed monocyte chimerism (45%) at 24 months maintaining good CH50 value and disease remission. In this group, 3 patients (P1, P6, P10) developed aGvHD of at least grade II. Two patients (P16 and P18) had Epstein-Barr Virus (EBV) reactivation with consequent development of post-transplant lymphoproliferative disorder (PTLD) in P18, both treated successfully with Rituximab.

After initial engraftment, three patients (P3, P4, P13), experienced secondary graft loss with a recurrence of autoimmunity. Two of them received a reduced intensity conditioning regimen, based on Treosulfan and Fludarabine in P3 and P4 and one received a reduced toxicity conditioning based on Busulfan (total dose received 177 mg/kg; target AUC 60–70 mg*h/L) and Fludarabine in P13.

P4 reached a chimerism of less than 20% 14 months after HSCT with a simultaneous drop of the C1q and CH50 levels and consequent recurrence of malar rash and oral ulcers that required further treatment with hydroxychloroquine and FFP infusions.

The first HSCT of P13 was complicated by grade III acute GVHD involving the skin and gut and by a severe autoimmune pancytopenia requiring treatment with steroids and immunosuppressors. After 146 days, she had secondary graft failure. Due to persistence of pancytopenia (considered as a possible manifestation of the underlying disorder), she underwent a second HSCT 9 months later achieving normalization of complement activity with initial improvement of pancytopenia. However, one month after the second procedure, she developed autoimmune haemolytic anaemia (likely transplant-related considering the persistence of 99% chimerism) that still requires treatment with steroids, cyclosporine, and Rituximab.

P3 had 7% chimerism 3 months after mismatched carrier related-donor HSCT. The nucleated cell dose in the graft was lower than desired (1.9 × 10^8/kg vs 3.0 × 10^8/kg as centre target dose). Despite initial normalization of classical complement function and disease control, 27 months after the HSCT she relapsed with CNS vasculitis (at that time the chimerism was 0%), requiring treatment with high dose of steroids, mycophenolate mofetil (MMF) and Rituximab. Considering the severity of the disorder, a second HSCT was attempted 4 years after the initial transplant. Despite establishing neutrophil engraftment, she developed progressive and irreversible respiratory failure secondary to aspergillus pneumonia and died 32 days after HSCT. Four other patients (P7, P9, P12 and P17) died after establishing neutrophil engraftment: a 13-year-old girl (P7), 3 months after HSCT, with multiorgan failure (MOF) secondary to transplant-associated thrombotic microangiopathy (TA-TMA), gastrointestinal GVHD (grade IV) and acute respiratory distress syndrome due to Methicillin-Resistant Staphylococcus Aureus (MRSA) pneumonia; a 16 year old girl (P9) with encephalopathy due to idiopathic hyperammonaemia after acute gastrointestinal GVHD (grade III); a 1 year old boy (P12) with respiratory failure secondary to cytomegalovirus (CMV) pneumonia; and a 9-year-old boy (P17), 4 months after HSCT, with MOF due to gastrointestinal acute GVHD (grade II) occurring after lymphocyte infusion for EBV-PTLD [20]. Of note, P3, P7 and P17 had a severe underlying disorder with neurologic involvement. Additionally, P7 had glomerulonephritis (grade IV) with active proteinuria and pulmonary hypertension at the time of HSCT.

At the time of HSCT, P9 was 16 years old and exhibited severe cutaneous and musculoskeletal involvement causing a very low performance status (Lansky score 30). After HSCT, she developed mood disorders with fluctuation in the level of consciousness secondary to idiopathic hyperammonaemia. At that time the chimerism was 95% (C1q and CH50 level not available). Due to the subsequent rapid deterioration of the neurological picture to death, cerebral magnetic resonance imaging (MRI) was not performed, and the cause of the encephalopathy remained undetermined. Underlying disease-related CNS involvement cannot be excluded given the absence of pre-transplant brain imaging.

Even though HSCT was performed at an early age before the development of organ damage, P12 died of CMV pneumonia. CMV serostatus was positive in the recipient and negative in the donor, and a CD34 + selected graft was used.

**Fig. 4:**
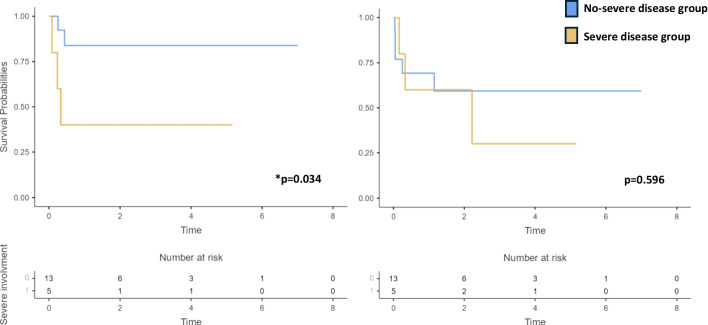
Comparison of overall survival and of event free survival between patients with severe and no-severe baseline disease. The presence of neurological and/or renal involvement were considered as markers of severe disease. Patients in severe group had worst OS (40% vs 84%; p = 0.034), while there was no difference in EFS at two years (60% vs 59%; p = 0.596) between the two groups. In the overall survival analys for the patients who had two HSCT, 2nd HSCT was considered as baseline

Overall, 5 patients (28%) had a baseline neurologic and/or renal involvement, both clinical markers of disease severity. As summarized in Fig. 4, the OS at 2 years in this subgroup was lower in comparison with the OS in the subgroup of patients without these complications (40% vs 84%; *p* = 0.034). We did not find any significant difference in the EFS between the two groups (60% vs 59%; *p* = 0.596)

## Discussion

Here, to our knowledge, we describe the largest cohort of C1q deficient patients treated with allogenic HSCT. Our findings strengthen previous case reports suggesting that HSCT may be a valid curative treatment, leading to restoration of the classical complement pathway, stable clinical remission and discontinuation of immunosuppressive treatments. In our cohort, the two-year OS was 71% and a long-term clinical response was obtained in 61% of patients. These data are comparable with a previous study on 128 patients with a large variety of severe autoimmune disorders treated with allogeneic HSCT, where OS was 70% at 5 years and 67% of patients reached a complete clinical response [25]. By contrast with this study, in which the non-relapse mortality was 21% at 5 years, in our cohort all deaths were transplant related.

In our case series, different kinds of donors were used, both matched and unmatched, as well as different conditioning regimens, including myeloablative and reduced toxicity protocols. Considering the limitation of the low number of patients it was not possible to find any clear correlation between donor type and conditioning regimen with HSCT outcome.

Given the improvement of outcomes in mismatched/haploidentical HSCT in patients with IEI, using TCR α-β depletion or post-transplant cyclophosphamide [26, 27], we could assume that these techniques may be a valid alternative approach also in C1q deficient patients in the absence of a well-matched donor. Indeed, among our cohort, one patient successfully underwent TCR α-β depleted transplant from a MMUD, with subsequent disease resolution, despite grade II aGvHD of the skin and HHV6 viraemia, successfully treated without any sequelae.

Due to the small numbers of patients, we cannot provide strong evidence on the impact of different conditioning regimens, but we could draw some provisional conclusions. First, we observed secondary graft failure in three patients after RTC in one and RIC in two of them, raising the question that a more robust conditioning may be needed to control the underlying immune-dysregulation and reach stable graft persistence. On the other hand, considering that mixed myeloid chimerism seemed to be sufficient to maintain disease control, a reduced intensity approach might be a valid option to minimize toxicity, as suggested in other IEI [28]. Further studies with larger sample sizes are needed to determine the best conditioning approach in these patients.

In terms of HSCT-related complications, three of four previously reported patients experienced post-transplant EBV reactivation, which resulted in PTLD in two. This raised concerns as to whether C1q deficient patients might be more susceptible to EBV reactivation [20–22]. We cannot confirm this association because no other cases of EBV reactivation were found in our cohort.

Perhaps due to the underlying immune dysregulation, we observed a high rate of inflammatory-mediated complications, with aGvHD of at least maximum overall grade II in 7 patients (39%) and development of haemolytic autoimmune anaemia in one patient. We speculate that an optimization of pre-transplant disease control, using for example specific bridging therapies (i.e. FFP, JAK inhibitors and type I interferon receptor blockade), may be helpful to achieve the best performance status before transplant, reducing the risk of transplant-related complications and graft failure.

As previously suggested, our review confirmed the variable clinical picture, with prominent mucocutaneous involvement associated with a significant percentage of neurological involvement. Renal disease and severe infections were less frequent in the transplant cohort in comparison with the larger cohort of reviewed cases.

As already reported in the literature, C1q deficiency can be associated with variable disease severity even within the same family, with some cases harbouring pathogenic biallelic mutations remaining asymptomatic throughout their lifetime [6, 7, 24]. In line with this, we did not find an association between different mutations and different patterns of clinical manifestations, perhaps consistent with a role of epigenetic and environmental factors as in SLE pathogenesis [29]. According to van Schaarenburg et al., mortality is estimated to be 20% before the age of 20 years [6]. However, it is important to interpret this finding with caution due to the possibility of an underestimation caused by the high number of cases lost to follow up, as well as an overestimation due to the presence of unrecognized patients.

Given these data, it is clear that a careful assessment of the risk and benefit of HSCT must be undertaken. On one hand, considering the related risk, HSCT should be considered only in patients where symptom control is not achievable with standard immunosuppressive treatments. On the other hand, it is important to transplant patients before the development of irreversible organ damage. Indeed, in our cohort, we showed that the OS after HSCT was worst in patients with severe autoimmune disease with extracutaneous involvement. In this regard, the definition of accurate predictors of disease severity would be helpful.

Triaille et al. have recently confirmed that C1q deficient patients demonstrate activation of the type 1 interferon pathway with elevated serum and cerebrospinal fluid levels of IFNα protein and an elevated expression of interferon-stimulated genes (ISGs) (a so-called interferon signature). Of note, ISG expression was corrected after HSCT in two patients who were evaluated here [23]. Thus, the evaluation of ISGs in blood might be a useful tool in patient assessment. In line with this concept, a higher ISGs expression has been reported to predict progression from ANA positivity to autoimmune connective tissue diseases in adult patients, thereby potentially allowing for risk stratification [30].

Based on the association between different autoantibodies and various clinical manifestations in rheumatic autoimmune disorders, we investigated if different auto-antibody profiles were associated with distinct organ involvement in C1q deficiency. We found that anti-Ro seems to be associated with neurological involvement, and anti-RNP and anti-DNA with renal involvement (although this result should be interpreted with caution due to lack of standardized measurements between different laboratories). The literature has already described levels of IFN strongly correlated with the levels of anti-Ro [12], thus supporting their possible role as markers of IFN dysregulation. Further larger prospective studies are needed to investigate the role of autoantibodies and interferon status in the assessment of patients with C1q deficiency, with the aim of early identification of patients at risk of severe disease, who may benefit from HSCT.

Given the rarity of the disease, this study is limited by the retrospective design, the small sample size, the wide heterogeneity of the disorder, transplant approaches and the limited follow-up. Moreover, due to the retrospective design, some patients had missing data regarding the length of GvHD prophylaxis and clinical details (such as definitive evidence of the autoimmune nature of cytopenias). However, the collective data that we report indicate that HSCT is a valid curative option in a specific subgroup of C1q deficient patients. In future, a more careful selection of patients and an optimization of HSCT, with possible use of therapies directly targeting IFNα (such as type I interferon receptor blockade and JAK inhibitors) as a “bridge to transplant”, may guide a tailored approach and to achieve improved outcomes.

## Supplementary Information

Below is the link to the electronic supplementary material.

Supplementary file1 (DOCX 17 KB)

## Data Availability

The data used in this study are not publicly available but may be available from the authors on reasonable request.

## Abbreviations

CH50: Classical complement pathway
CMV: Cytomegalovirus
CNS: Central nervous system
EBV: Epstein-Barr virus
EFS: Event free survival
FFP: Fresh frozen plasma
GvHD: Graft versus host disease
HSCT: Hematopoietic stem cell transplantation
IFN-α: Interferon-alfa
MALT: Mucosa-associated lymphoid tissue
MMF: Mycophenolate mofetil
MMRD: Mismatched related donor
MMUD: Mismatched unrelated donors (< 10/10)
MOF: Multiorgan failure
MSD: Matched sibling donors
MRI: Magnetic resonance imaging
MRD: Matched related donors
MRSA: Methicillin-resistant Staphylococcus Aureus
MUD: Matched unrelated donors
OS: Overall survival
PBSC: Peripheral blood stem cells
PTLD: Post-transplant lymphoproliferative disorder
SLE: Systemic lupus erythematous
TMA: Transplant-associated thrombotic microangiopathy
