# Supplementary material for: Hematopoietic Stem Cell Transplantation for C1q Deficiency: A Study on Behalf of the EBMT Inborn Errors Working Party
Source: J Clin Immunol. 2024 Oct 29;45(1):35. doi: 10.1007/s10875-024-01819-1 (PMC11522153; doi:10.1007/s10875-024-01819-1)
Supplement: Supplementary file 1 — Supplementary file1 (DOCX 17 KB) [file 10875_2024_1819_MOESM1_ESM.docx]

***Supplementary table 1. Patient C1Q and CH50 level before and after HSCT***

|  | ***C1Q level*** | | | ***CH50*** | | |
| --- | --- | --- | --- | --- | --- | --- |
| ***pt.*** | ***Pre-HSCT*** | ***After HSCT*** | ***Normal range*** | ***Pre-HSCT*** | ***After HSCT*** | ***Normal range*** |
| *P1* | *Undetectable* | *187 mg/L* | *50-250 mg/L* | *15.7 U/mL* | *90.4 U/mL* | *41.7-95.1U/ml* |
| *P2* | *Undetectable* | *146 mg/L* | *50-250 mg/L* | *NA* | *46.8 U/mL* | *41.7-95.1U/ml* |
| *P3 (1^st^)* | *Undetectable* | *Undetectable* | *50-250 mg/L* | *NA* | *Normalization* | *41.7-95.1U/ml* |
| *P3(2^nd^)* | *Undetectable* | *NA* | *50-250 mg/L* | *NA* | *NA* | *41.7-95.1U/ml* |
| *P4* | *24.45 mg/L* | *72 mg/L* | *147-179 mg/L* | *6.8 U/mL* | *42.09 U/mL* | *144-240 U/mL* |
| *P5* | *Undetectable* | *57 mg/L* | *50-86 mg/L* | *Undetectable* | *81 U/mL* | *41.7-95.1 U/mL* |
| *P6* | *Undetectable* | *111 mg/L* | *102-171 mg/L* | *Undetectable* | *114%* | *>74%* |
| *P7* | *NA* | *NA* | */* | *Reduced* | *NA* | */* |
| *P8* | *20 mg/L* | *140 mg/L* | *120-220 mg/L* | *Undetectable* | *90 U/mL* | *70-150 U/mL* |
| *P9* | *NA* | *NA* | */* | *Undetectable* | *NA* | */* |
| *P10* | *NA* | *NA* | */* | *Undetectable* | *NA* | */* |
| *P11* | *112 mg/L* | *NA* | *>120 mg/L* | *Undetectable* | *84 U/mL* | *70-150 U/mL* |
| *P12* | *Undetectable* | *144 mg/L* | *102- 171 mg/L* | *NA* | *190 CAE* | *60-144 CAE* |
| *P13(1^st^)* | *Undetectable* | *NA* | *118-244 mg/L* | *Undetectable* | *NA* | *41-95 U/mL* |
| *P13(2^nd^)* | *NA* | *85 mg/L* | *118-244 mg/L* | *NA* | *88 U/mL* | *41-95 U/mL* |
| *P14* | *Undetectable* | *127 mg/L* | *88-153 mg/L* | *Undetectable* | *59.9 U/mL* | *30-46 U/mL* |
| *P15* | *28 mg/L* | *106 mg/L* | *88-153 mg/L* | *Undetectable* | *40.4 U/mL* | *30-46 U/mL* |
| *P16* | *Undetectable* | *82 mg/L* | *70-140 mg/L* | *Undetectable* | *659 U/mL* | *392-1019 U/mL* |
| *P17* | *Undetectable* | *40%* | *78-131%* | *Undetectable* | *80%* | *65-125%* |
| *P18* | *Undetectable* | *40%* | *78-131%* | *Undetectable* | *100%* | *65-125%* |

Abbreviations: NA not available
